# Pneumonia Inoculation

**Published:** 1916-04

**Authors:** 


**Pneumonia Inoculation.** F. S. Lister, Med. Jour. of S. Africa, Nov., 1915, finds that three, subcutaneous injections of 100-400 million killed pneumococci do not develop demonstrable agglutinins nor opsonins in the serum of rabbits but that a single intravenous injection of 100 million does. Intravenous injection of weak vaccine of 5 different strains, in a human being, develops the specific agglutinins and opsonins for each strain.

